# Treatment of Catarrh

**Published:** 1879-03-20

**Authors:** W. F. Duncan


### *TREATMENT OF CATARRH.*

BY W. F. DUNCAN, M.D.

The successful treatment of catarrh is largely confined to local applications, although the necessity for treating internally every disorder of the system is earnestly urged. Always in treating a diseased surface, cleanliness is recognized as the chief requisite. This necessity, I repeat, is especially emphasized in dealing with a diseased mucous membrane, which must be thoroughly cleansed before the application of medicine is made. The mucus is often very tenacious, and secreted in cavities difficult of access, and yet it is possible to remove most of it by the methods described. The fact that alkaline solutions have a solvent effect on mucus is utilized, and all of the cleansing solutions contain some form of alkali; and as in many cases there is a decomposition of the retained secretion, an antiseptic or disinfectant is used. Any combination of these two medicines, in weak solution, will answer, but that which seems to be as efficient as any, and in use at the clinic, is Dobell's solution.

℞ Acidi carbol.............. ʒ iss.

Sodii biboratis,

Sodii bicarb:, aa.......... ʒ ij.

Glycerinæ................ f. ℥ ij.

Aquæ ad.................. f. Oij. M.

It is used with the atomizer, the post-pharyngeal syringe, and the nasal douche. The nasal douche of Thudichum has received too much praise and too much condemnation. It has a position in the armenture worthy of a moment's consideration. When a catarrh is simple, there is nothing but an excess of secretion, and it is limited to the anterior nares, the use of nasal douche is serviceable. It is valueless in any other case, however, because the solution washes only a limited surface. It enters one nostril, and, flowing upward around the rear of the septum, passes out of the other, cleansing only the inferior meatuses, and does not reach the whole of the vault. Again, it does not run with sufficient force to be of much value when there is a copious sticky secretion. There is some danger to be apprehended from the solution entering the Eustachian tubes, beyond the valvular portion, if used carelessly. This liability is reduced to a mere nothing if the patient be directed to hold the nose downwards, and while the current is passing through the nostrils to breath through the open mouth. Also the vessel or reservoir must not be placed more than two fee: above the level of the head. Common salt ʒ i.—aq. Oi. may be of service. I have abandoned the douche because of its limited service, except when used with a curved nozzle, like the pipe of the post-pharyngeal syringe, which is past behind the soft palate, and the solution runs out of both nostrils. I recommend this to be used by the patient at his home.

The best method of using the cleansing solution is with the post-pharyngeal syringe, which is both safe and efficient. The solution can be driven with a great deal of force without danger of its entering the middle ear, because the direction of the stream and the Eustachian tubes is the same, downwards and forwards. It is to be entered flat on the tongue, which is depressed by its nozzle, its point introduced quickly behind the palate, and the contents suddenly and forcibly ejected by driving home the piston, and the syringe withdrawn. When there are crusts and plugs of mucus, it may be necessary to repeat its use a dozen or more times at a sitting before they are washed away. Always examine to see that the surface is clean. When skillfully used, it gives no pain, and is tolerated by any patient. Sometimes the sticky pellicle in ozæna will be loosened and drawn down from the upper meatuses until it reaches the anterior nares, where it will remain. It can be dislodged by throwing a stream with the same syringe, first into the nares in front, and then from behind the palate. The solution can also be used in a spray driven by compressed air, either by a hand-ball atomizer, or a pump and receiver. The last is ve[illegible] efficient when used with about thirty (30) pounds pressure, and will dislodge mucus from the superior meatuses, and even the entrance of the sinuses. It is better for children than the post-pharyngeal syringe. If, with all these methods, you fail to clear the nostrils, as you may do in syphilis, loosen the crusts with a probe, and remove them with long, slender forceps.

The next step in the treatment is the application of the medicines adapted to the case, which is made in the form of spray, powder or solution. The spray spreads out in every direction, and reaches cavities otherwise almost inaccessible, and is, therefore, the choice method. In simple catarrh the object in view is to reduce the amount of inflammation by the use of astringents. Select astringents of different strengths and kinds to suit each case. For a standard astringent, sulphate of zinc, gr. xv.—aq. ʒ j., is a good one. If the case be a mild one do not use it stronger than three grains. If the catarrh be of long standing see the patient three times a week, and in the intervals let him use the cleansing solution home, with Delano's atomizer, or the post-pharyngeal douche. Ferric-alum, gr. v.—xx. to aq. ℥j., is valuable when there is excess of secretion and little sensibility. Chlorate of potash, nitrate of silver, tannin and chloride of zinc may be used. Ring the changes on the astringents until a good one is found, and stick to it. When pain, lasting longer than half an hour, follows the use of the astringent, use a spray of U. S. solution of morphine. When there is hypertrophy to deal with, stronger applications are needed. Caustics can be applied with a probe, one end of which is tightly wrapped with cotton. With such a probe, one end of which is bent at right angles, the short arm of which is about an inch long, applications can be made behind the palate to the vault. The hypertrophied tissue may be destroyed, crushing it with forceps, cutting it with a knife, and galvano-cautery are allowable. The polypoid thickening of the ends of the turbinate bones can be touched with caustics, applied by means of a probe passed through a shield. Curette the vault when there is adenoid degeneration. In both the above forms of catarrh excess of secretion is the prominent feature requiring treatment.

In the atrophic form the secretion is absent, and the glands need to be stimulated to action, and astringents avoided. A spray from a weak solution of iodine, gtt. v.—x. to aq. ℥i., or tr. sanguinaria ʒ i. to aq. ʒ i., may be used. Sang., myrrh, and lycopodium in powder, blown into the nostrils, are a valuable stimulant. Continued applications to a perfectly dry membrane bring a reward after a time, when the stumps of the glands begin to take on action and pour out the secretion.

The simple ozæna is treated by carefully removing the pellicle every day or two, and then using an astringent spray, after which idioform blown into the nostrils in powder is effective. The nasal passages must constantly be kept open so as to allow all the offensive matter to flow freely out of the accessory cavities. The idioform is not annoying to the patient, and, if care be taken not to get any of it on the clothing, will not be very disagreeable to others. When syphilitic ozœna exists the local treatment is the same. In addition, the usual internal remedies are employed. If any dead bone can be detached take it away at once. Finally, take up each complication singly and overcome it, remove all foreign bodies and tumors, fight every disease and diathesis with the proper remedies, and the same measure of success will be met with in treating catarrh as is encountered in treating other chronic disorders.—*N. Y. Med. Record.*

